# Balancing Molecular Sensitization and Surface Passivation in Lanthanide-Doped Nanoparticle-Based Organic–Inorganic Nanohybrids

**DOI:** 10.1021/acs.nanolett.5c04324

**Published:** 2025-10-28

**Authors:** Zhao Jiang, Alasdair Tew, Xinjuan Li, Huangtianzhi Zhu, Yunzhou Deng, Caterina Ducati, Zhongzheng Yu, Akshay Rao

**Affiliations:** ‡ Cavendish Laboratory, 2152 University of Cambridge, Cambridge CB3 0HE, United Kingdom; § Department of Materials Science and Metallurgy, 2152 University of Cambridge, Cambridge CB3 0FS, United Kingdom

**Keywords:** lanthanide-doped nanoparticle, molecular sensitization, surface passivation, triplet energy transfer

## Abstract

Lanthanide-doped nanoparticles (LnNPs) are promising for advanced photonic applications due to their unique optical properties. However, their practical implementation is hindered by surface quenching and weak absorption. Surface passivation through core–shell architectures is effective in mitigating quenching. However, it creates a fundamental trade-off by impeding molecular sensitization via energy transfer (ET) in the organic–inorganic hybrid systems. Here, we investigate this trade-off by fabricating core–shell LnNPs with precisely controlled shell thicknesses ranging from 0.8 to 3.0 nm. Surface passivation yields enhancements in 290-fold upconversion intensity and 25-fold downshifting intensity. Using 9-anthracenecarboxylic acid, we demonstrate that ET efficiency exhibits a nonmonotonic dependence on the shell thickness, with optimal performance achieved at a shell thickness of ∼0.8 nm. Through steady-state and time-resolved spectroscopic studies, we elucidate the complex ET dynamics. Our findings reveal the optimal shell thickness and answer whether no shell is the best in this nanohybrid system.

## Graphical abstract

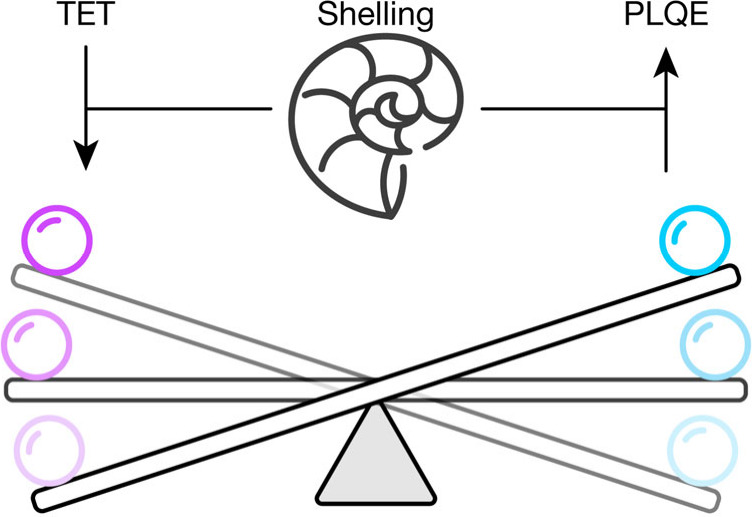

Lanthanide-doped nanoparticles (LnNPs) possess many desirable properties, including narrow emission bands, long-lived luminescence, and high photostability, making them highly versatile for applications such as upconversion,^1–4^ laser technology,^5–7^ infrared detection,^8^ imaging,^9,10^ optogenetics,^11,12^ and encryption.^13,14^ However, despite their promise, LnNPs face two major challenges: surface quenching and weak absorption.

Surface quenching arises primarily from two sources. First, surface defects in nanocrystals serve as quenching centers, introducing nonradiative relaxation pathways that dissipate excitation energy.^15^ Second, surface adsorbates, such as water or organic contaminants, facilitate nonradiative deexcitation of lanthanide ions via vibrational coupling with the lanthanides’ abundant electronic energy levels.^16^ To address these issues, surface passivation has emerged as an effective strategy to mitigate quenching and improve nanoparticle (NP) stability.^17,18^ By reducing surface defects and minimizing interactions with environmental molecules, surface passivation not only decreases nonradiative energy losses but also protects the excited states of lanthanide ions, thereby enabling high luminescence efficiency. Due to this effectiveness, core–shell LnNP architectures, typically employing shells of NaLnF_4_, CaF_2_, or ScF_3_, have become a standard design for achieving efficient lanthanide emitters.^19–22^ A sufficiently thick shell, often exceeding 5 nm, is critical for effective passivation, yielding large luminescence enhancements compared to unshelled materials.^17,23^

Another significant limitation of LnNPs is their inherently weak absorption, a consequence of the parity-forbidden nature of f–f electronic transitions within the 4f orbitals, as dictated by the Laporte rule.^24^ This restriction results in absorption coefficients that are often 6 orders of magnitude lower than those of organic chromophores, significantly reducing their photoluminescence quantum efficiency (PLQE) and brightness.^25,26^ To overcome this, sensitization with organic molecules has been widely employed.^27–29^ These molecules act as antennas, efficiently absorbing photons and transferring energy to Ln^3+^ ions. Energy transfer between molecules and LnNPs occurs via two main mechanisms. Förster resonance energy transfer (FRET) operates through dipole–dipole coupling, enabling the transfer of spin-zero singlet excitons by coupling to the absorption dipole of the Ln^3+^ ion.^30,31^ However, the weak oscillator strength of Ln^3+^ ions makes them poor energy acceptors in this process.

More recently, it has been shown that Dexter-like triplet energy transfer (TET) from triplet excitons on organic chromophores to Ln^3+^ ions doped in LnNPs can occur on fast time scales and with near-unity efficiency.^32–34^ This process relies on fast intersystem crossing (ISC) facilitated by spin-exchange coupling with the unpaired electrons on the lanthanide ion.^32–35^ The high efficiency of this process opens up new pathways for the control of triplet excitons and sensitization of LnNPs. However, normally TET requires subnanometer proximity between donor and acceptor sites, due to the necessity of orbital overlap within this tunnelling process. This strict dependency creates a fundamental trade-off: thick passivation shells enhance luminescence efficiency but shut down TET-based molecular sensitization. Despite the significance of this interplay, systematic studies on how the shell thickness influences surface passivation and energy-transfer dynamics remain scarce, leaving a critical gap in the field.

Figure 1a shows the model molecular sensitizer, 9-anthracenecarboxylic acid (ACA), and core-inert shell NaGdF_4_:Yb_0.2_,Er_0.02_@NaGdF_4_ NPs that we have chosen in this study. We systematically investigated the relationship between shell passivation and molecular energy transfer in LnNP–molecule hybrids as a function of the shell thickness (Figure 1b). The inert shell thicknesses were precisely controlled, ranging from 0.8 to 3.0 nm. Shell passivation results in nearly linear enhancements in the upconversion emission (up to 290-fold), downshifting emission (up to 25-fold), and Er^3+^ lifetime (up to 12-fold) at 1530 nm. The LnNP-ACA hybrids are fabricated via partial ligand exchange. Through comprehensive steady-state and time-resolved spectroscopic investigations, we demonstrate that the hybrid system exhibits optimal energy-transfer efficiency at an intermediate shell thickness of ∼0.8 nm, where surface passivation benefits are maximized while maintaining efficient TET. We further employ transient absorption (TA) spectroscopy to elucidate the temporal dynamics of energy-transfer processes, revealing the complex interplay between the ISC, energy-transfer, and surface quenching mechanisms. Our findings establish design principles for optimizing the energy transfer–surface passivation trade-off in lanthanide-doped core–shell nanostructures, providing guidance for developing hybrid nanomaterials with tailored photophysical properties.

**Figure 1:**
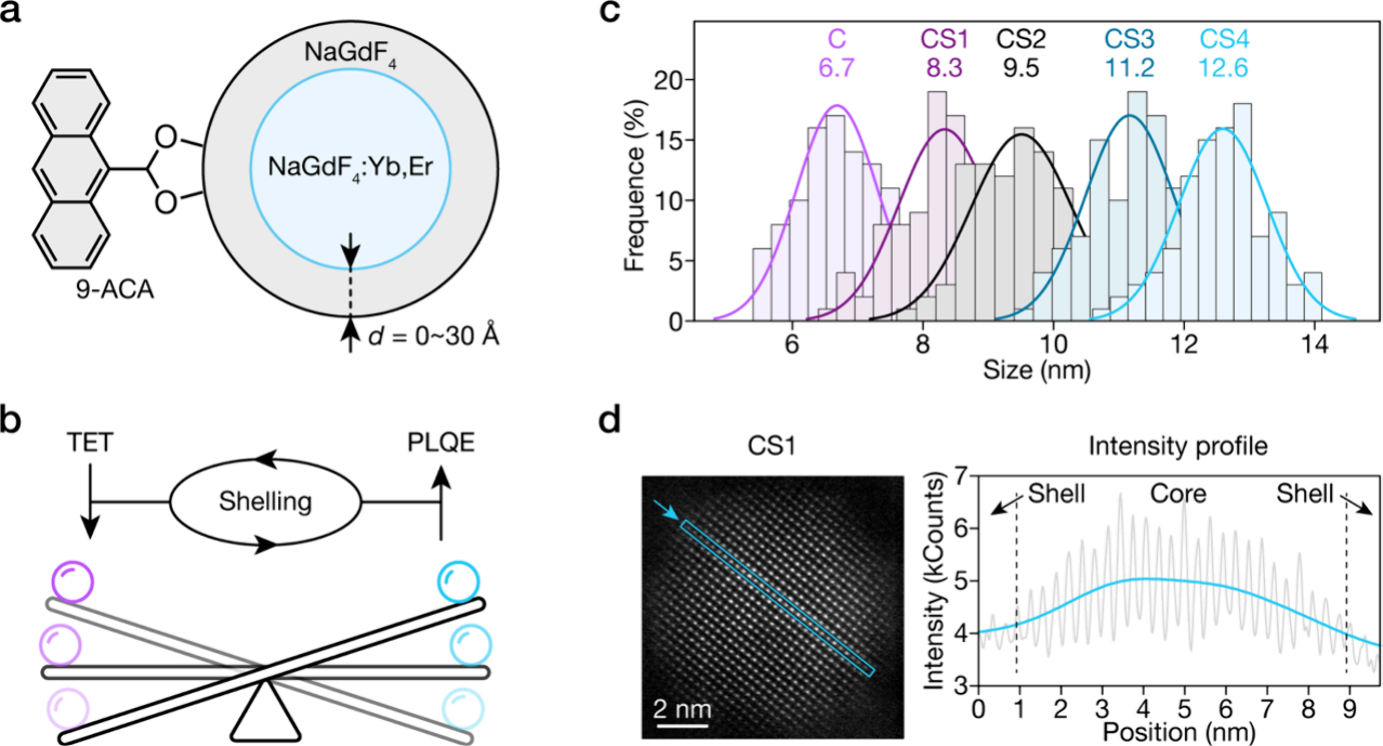
(a) Schematic illustration of the architecture of the LnNP-ACA hybrids. (b) Schematic illustration showing the paradoxical relationship during shell growth: increasing shell thickness leads to enhanced PLQE of LnNPs but decreasing efficiency of TET between the organic ligand and lanthanide ions. (c) Statistical histogram of LnNP size distributions for core (C) and core–shell (CS1–CS4) NPs, showing progressive size increases corresponding to increasing shell thickness across the series. (d) HAADF-STEM image of the CS1 sample (left panel) and its corresponding intensity profile (right panel) along the blue arrow.

The NaGdF_4_:Yb_0.2_,Er_0.02_ core NPs were synthesized using a coprecipitation method (see the Supporting Information).^36^ The core NPs were then passivated through epitaxial growth of an undoped NaGdF_4_ shell using a high-temperature hot-injection technique (see the Supporting Information).^37^ Epitaxial shell growth was controlled to maintain crystallographic alignment with the core, minimizing lattice mismatch at the interface. By varying the amount of injected shell precursor, we obtained NaGdF_4_:Yb_0.2_,Er_0.02_@NaGdF_4_ core–shell NPs with tunable shell thickness (Figure S1). Figure 1c shows statistical analysis of the transmission electron microscopy (TEM) images, showing increasing average particle size from 6.7 to 12.6 nm for samples from C to CS4, respectively. The progressive diameter increases across the series with narrow size distributions, as mapped out via TEM, confirming our excellent shell growth control.

To further investigate the core–shell structure, high-angle annular dark-field scanning transmission electron microscopy (HAADF-STEM) was performed on CS1. As shown in Figure 1d, the image reveals the crystalline lattice structure with high atomic resolution. The intensity profile across the particle (blue arrow) shows a characteristic pattern with decreasing intensity at the outer ring, confirming the distinct core–shell structure.

The passivation effect of the NaGdF_4_ shell on NaGdF_4_:Yb_0.2_,Er_0.02_ core NPs was systematically investigated by analyzing upconversion luminescence, downshifting emissions, and Er^3+^ lifetimes as a function of the shell thickness. To ensure a quantitative comparison across samples with varying shell thicknesses, we standardized the particle concentration by measuring the characteristic Yb^3+^ absorption at 980 nm (Figure S2). Because Yb^3+^ ions are located exclusively in the core and the NaGdF_4_ shell is optically inactive in this region, equivalent Yb^3+^ absorption confirms identical particle densities across samples. As shown in Figures 2a and S3, even a thin 0.8 nm shell (CS1) produced a remarkable 26-fold enhancement in the upconversion emission compared to the unshelled core (C), highlighting the extreme sensitivity of upconversion to surface quenching of codoped LnNPs. Beyond this initial passivation, the emission intensity increased linearly with the shell thickness across samples CS1–CS4. The upconversion spectra reveal characteristic Er^3+^ emission bands at 410 nm (^2^H_9/2_–^4^I_15/2_), 525 nm (^2^H_11/2_–^4^I_15/2_), 542 nm (^4^S_3/2_–^4^I_15/2_), and 660 nm (^4^F_9/2_–^4^I_15/2_), with the green emission at 542 nm exhibiting the most pronounced enhancement. This enhancement is quantified in Figure 2b, where the upconversion luminescence enhancement factor increases linearly with a slope of 12.1/Å. The steep slope underscores the effectiveness of spatial isolation in preserving energy-transfer pathways for upconversion. Notably, enhancement shows no saturation even at the thickest shell (CS4, 3.0 nm), indicating that complete passivation of core NPs has not yet been achieved.

**Figure 2:**
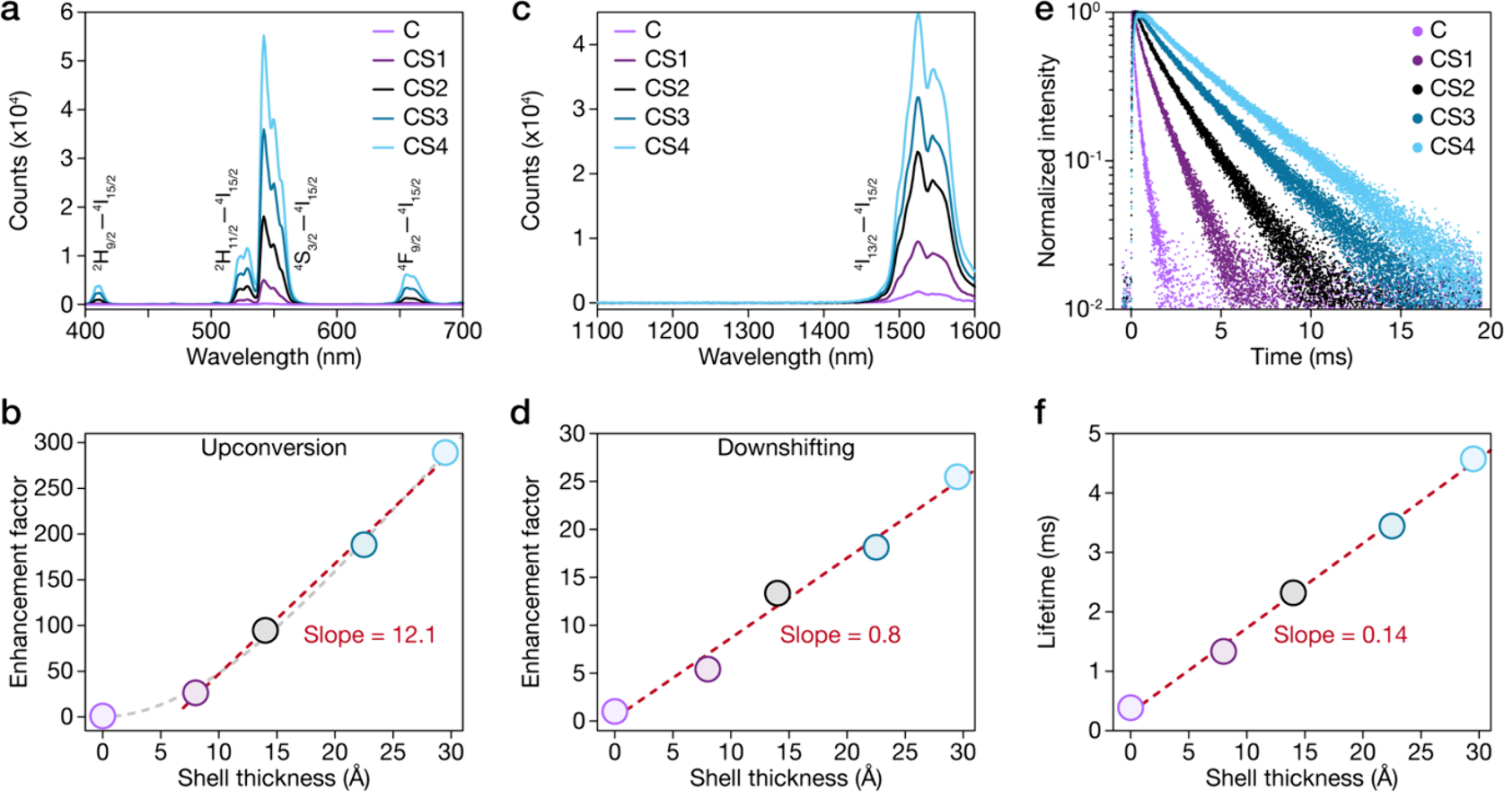
(a) Upconversion emission spectra of core (C) and core–shell (CS1–CS4) LnNPs under 980 nm excitation with progressive intensity enhancement as the shell thickness increases. (b) Quantitative analysis of the upconversion PL enhancement factor versus shell thickness. (c) Downshifting NIR emission spectra of LnNPs under 980 nm excitation with the systematic intensity increase correlating with the shell thickness. (d) Quantitative analysis of downshifting PL enhancement factor versus shell thickness. (e) Time-resolved PL decay curves of LnNPs monitored at 1530 nm (Er^3+4^I_13/2_–^4^I_15/2_ transition) under 980 nm excitation. (f) Fitted luminescence lifetimes extracted from decay curves in part e plotted as a function of the shell thickness.

Similar enhancement was observed for Er^3+^ downshifting near-infrared (NIR) emission at 1530 nm (^4^I_13/2_–^4^I_15/2_) under 980 nm excitation (Figure 2c). The NIR emission intensity increased progressively with the shell thickness, confirming the beneficial effect of surface passivation on both upconversion and downshifting luminescence. However, the enhancement factor for downshifting emission (Figure 2d) exhibited a significantly lower slope of 0.8/Å compared to upconversion (12.1). This 15-fold difference in enhancement factors provides critical insights into the underlying photophysical mechanisms. Upconversion luminescence, which relies on sequential photon absorption and complex energy-transfer processes between lanthanide ions (Yb^3+^→Er^3+^), is inherently more vulnerable to surface quenching. The multistep nature of upconversion requires long-lived intermediate states that are easily quenched through nonradiative pathways at the surface. In contrast, downshifting emission primarily involves a single excitation–emission cycle, making it less dependent on interionic energy transfer and less susceptible to surface quenching. This mechanistic difference explains the higher sensitivity of upconversion to shell thickness and confirms that surface passivation predominantly enhances processes involving multiple energy-transfer steps and long-lived excited states.

Time-resolved luminescence measurements provide additional evidence for the passivation effect. Figure 2e presents the PL decay curves of Er^3+^ emission at 1530 nm under 980 nm excitation for all samples. A progressive deceleration of decay kinetics with increasing shell thickness is clearly observed, indicating effective suppression of nonradiative decay pathways. The decay curves were fitted to calculate the luminescence lifetimes, which are plotted against the shell thickness in Figure 2f. Lifetime increases linearly from 0.4 ms for the unshelled core to 4.6 ms for the CS4 sample (3.0 nm shell) with a slope of 0.14 ms/Å, representing an 11.5-fold extension. The linear relationship confirms that passivation continues to increase even for the thickest shell investigated, suggesting optimal passivation require shells thicker than 3.0 nm. This lifetime extension provides mechanistic insight into the passivation effect. The NaGdF_4_ shell protects the Er^3+^ excited state by inhibiting nonradiative decay channels: (1) eliminating high-frequency vibrational coupling to surface ligands and solvent molecules that accelerate multiphonon relaxation; (2) minimizing energy transfer to surface defects acting as quenching centers; (3) reducing cross-relaxation pathways between surface-proximal lanthanide ions that depopulate excited states; (4) modifying the local crystal field environment, potentially enhancing radiative decay rates relative to nonradiative processes.

These results confirm that surface passivation through epitaxial shell growth significantly enhances the optical properties of codoped LnNPs. The linear relationships between the optical parameters (emission intensity and lifetime) and shell thickness indicate proportional reduction in nonradiative decay pathways with increasing spatial isolation. The continuing enhancement at the maximum shell thickness of 3.0 nm suggests that complete passivation would require even thicker shells, consistent with previous reports on similar core–shell systems.^16,18^ This understanding of shell-thickness-dependent passivation provides guidance for the rational design of organic–LnNPs hybrids we develop below.

In organic–inorganic hybrid systems, emission brightness depends on absorption cross-section, energy-transfer efficiency, and PLQE. Building upon our investigation of shell-thickness-dependent optical properties, we explored TET kinetics to understand how the shell thickness affects the energy-transfer efficiency in these competing processes. The LnNP-ACA hybrids were fabricated by mixing oleic acid-capped LnNPs in hexane with 9-ACA solution, followed by stirring at room temperature and purification to remove uncoordinated ligands (see the Supporting Information). The absorption spectra of the LnNP-ACA hybrids (Figure S4) confirmed successful ligand exchange, showing significantly stronger absorption than the pristine LnNPs. The excitation spectra of pristine LnNPs monitored at Er^3+^ emission (1530 nm) are presented in Figures 3a and S5. These spectra exhibit characteristic narrow bands corresponding to direct 4f–4f transitions of Er^3+^ ions, with prominent peaks at 377 nm (^4^I_15/2_ → ^4^G_11/2_), 407 nm (^4^I_15/2_ → ^2^H_9/2_), 488 nm (^4^I_15/2_ → ^4^F_7/2_), and 521 nm (^4^I_15/2_ → ^2^H_11/2_). The intensity of these excitation bands increases systematically with the shell thickness, consistent with enhanced luminescence due to surface passivation.

**Figure 3:**
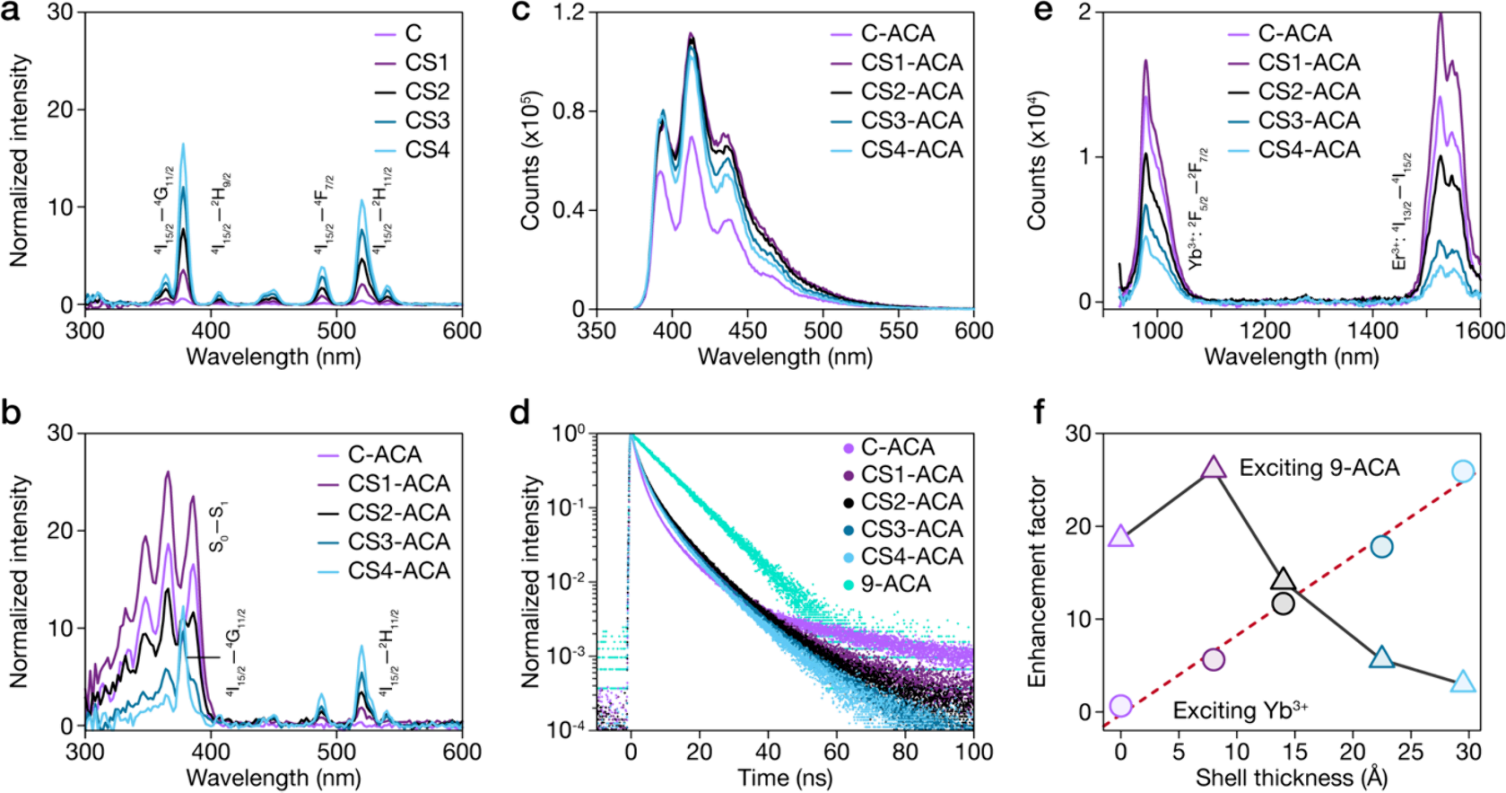
(a) Excitation spectra of pristine core (C) and core–shell (CS1-CS4) LnNPs monitored at Er^3+^ emission (1530 nm). (b) Excitation spectra of LnNP-ACA hybrids monitored at Er^3+^ emission (1530 nm). (c) Visible emission spectra of LnNP-ACA hybrids under 366 nm excitation showing characteristic vibronic emission of surface-attached ACA molecules. (d) Time-resolved PL decay curves of LnNP-ACA hybrids and free ACA monitored at 410 nm under 375 nm laser excitation. (e) NIR emission spectra of LnNP-ACA hybrids under 366 nm excitation. (f) PL enhancement factors for emissions at 1530 nm plotted as a function of the shell thickness.

Attached with ACA, the excitation spectra of these hybrids align well with the absorption spectra of ACA (Figures 3b and S5). This spectral change provides direct evidence for energy transfer from surface-bound ACA molecules to LnNPs, demonstrating effective sensitization of NIR emission through organic molecules. However, the sensitization efficiency exhibits complex dependence on the shell thickness. For thin-shelled samples (C-ACA and CS1-ACA), the ACA contribution is particularly prominent, suggesting efficient energy transfer across the core–shell interface. As the shell thickness increases beyond 0.8 nm (CS1), the contribution from ACA band progressively diminishes, indicating attenuated energy transfer with increasing donor–acceptor separation. Concurrently, intrinsic Er^3+^ excitation peaks become more pronounced with increasing shell thickness, reflecting enhanced contribution from lanthanide absorption. The sharp peak at 377 nm in thicker-shelled samples (CS3-ACA and CS4-ACA) should be attributed to the ^4^I_15/2_ → ^4^G_11/2_ transition of Er^3+^ rather than enhanced ACA contribution. This peak becomes more resolved as the shell thickness increases due to reduced spectral overlap with the diminishing ACA contribution, further evidencing the distance-dependent nature of TET. This assignment is supported by the distinct spectral characteristics that ACA exhibits typical broad π–π* absorption (300–400 nm), while the Er^3+^ 4f–4f transition appears as a narrow line characteristic of parity-forbidden lanthanide transitions.

The visible emission spectra of LnNP-ACA hybrids under 366 nm excitation (Figure 3c) predominantly feature the characteristic vibronic emission structure of ACA, with peaks between 380 and 500 nm. Time-resolved fluorescence measurements of ACA emission at 410 nm under 375 nm excitation (Figure 3d) reveal considerable change to the chromophore’s excited-state dynamics upon attachment to LnNPs. While free ACA in solution exhibits single-exponential decay kinetics with a monoexponential lifetime of 9.53 ns, all LnNP-ACA hybrids display complex biexponential decay profiles. Quantitative biexponential fitting (Table S1) reveals distinct fast (τ_1_) and slow (τ_2_) decay components for all hybrid samples. The C-ACA sample exhibits the most dramatic changes, with τ_1_ = 1.66 ns (77.34% contribution) and τ_2_ = 7.25 ns (22.66% contribution), representing substantial acceleration compared to free ACA. The biexponential decay reflects spatial heterogeneity of ACA molecules on the NP surface. The dominant fast component corresponds to ACA molecules well-coupled to Gd^3+^/Yb^3+^/Er^3+^ ions with efficient energy transfer. The minor slow component represents molecules at surface sites with hindered energy transfer due to suboptimal binding geometry or coordination at defect sites, retaining lifetimes closer to intrinsic radiative decay. This significant lifetime reduction provides direct kinetic evidence for efficient energy transfer from excited ACA to lanthanide ions in the unshelled core, where the proximity enables rapid nonradiative energy transfer. As the shell thickness increases, both decay components systematically increase but are still shorter than the lifetime of ACA. This indicates the distance-dependent modulation of TET efficiency.

The NIR emission spectra of LnNP-ACA hybrids under 366 nm excitation (Figure 3e) reveal two prominent bands centered at 980 and 1530 nm, corresponding to the ^2^F_5/2_ → ^2^F_7/2_ transition of Yb^3+^ and the ^4^I_13/2_ → ^4^I_15/2_ transition of Er^3+^, respectively. The simplified energy-transfer scheme has been shown in Figure S6. A striking feature is the nearly 1:1 intensity ratio between Yb^3+^ and Er^3+^ emission bands across all samples, despite significant variation in shell thickness. This consistent ratiometric emission suggests that the Yb^3+^-to-Er^3+^ energy-transfer efficiency remains largely unaffected by shell growth, as this process occurs primarily within the core where dopant distribution remains unchanged. Instead, overall intensity variation across samples predominantly reflects modulation of the initial ACA-to-Yb^3+^ energy-transfer step by shell thickness. The maximum overall NIR emission intensity is obtained in CS1-ACA hybrids.

To explain the reason why CS1-ACA yields the best NIR PL performance, we compared Er^3+^ emission at 1530 nm under two excitation conditions, i.e., direct Yb^3+^ excitation at ∼980 nm and molecular sensitization through ACA at 300–400 nm (Figure S5). Figure 3f reveals different trends that illuminate the energy transfer–surface passivation trade-off. Under direct Yb^3+^ excitation, Er^3+^ emission increases monotonically with shell thickness, reaching ∼25-fold enhancement for CS4-ACA. This reflects progressive surface passivation, as previously observed in Figure 2d. In contrast, molecular sensitization through ACA excitation exhibits pronounced nonmonotonic behavior, peaking at CS1-ACA with ∼26-fold enhancement and then declining to ∼3-fold for CS4-ACA. This bell-shaped dependence exemplifies the fundamental energy transfer–enhancement paradox (Figure 1b). For C-ACA, despite optimal proximity for energy transfer, severe surface quenching limits sensitized emission. CS1-ACA achieves best performance by balancing surface passivation with efficient energy transfer. Beyond this optimal thickness, progressive spatial separation exponentially attenuates energy-transfer efficiency, causing sensitized emission to decline despite continued improvement in LnNPs passivation. The identification of the optimal shell thickness (∼8 Å) provides reference for lanthanide–organic hybrid systems, establishing the length scale where energy-transfer efficiency and surface passivation achieve optimal balance.

The whole energy-transfer process for the codoped LnNP-ACA hybrids is complicated, involving energy transfer from ACA to Yb^3+^ and Er^3+^, radiative emission from Yb^3+^, energy transfer from Yb^3+^ to Er^3+^, and the radiative emission of Er^3+^ (Figure S6). The energy transfer from ACA to LnNPs involves efficient TET from the triplet excitons to Yb^3+^ and less efficient FRET from the singlet emission to Er^3+^ or Yb^3+^. Thus, this system is more complicated than the single dopant LnNPs. To gain deeper mechanistic insights into the complicated energy-transfer processes, we performed TA spectroscopy studies on the LnNP-ACA hybrid systems (Figure 4 and Table S2).

**Figure 4:**
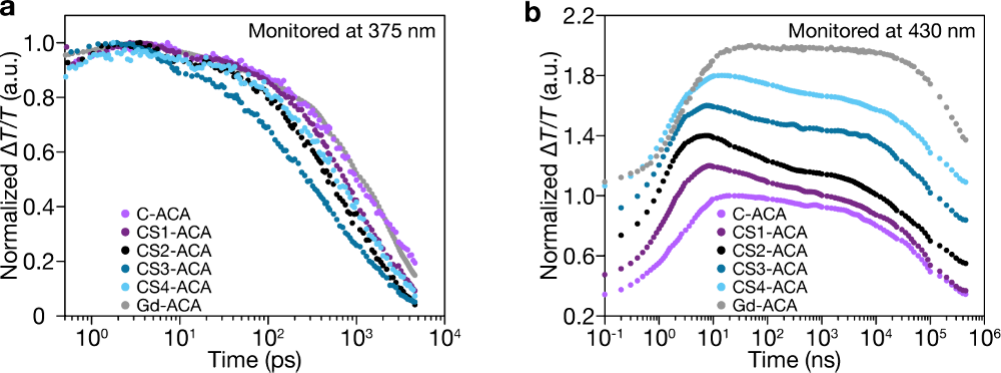
(a) Picosecond TA traces of normalized singlet signals (375 nm). (b) Nanosecond TA traces of normalized triplet signals (430 nm).

Figure 4a presents normalized picosecond TA data for the control GdNPs, core and core–shell samples, monitoring singlet exciton signal at 375 nm. Compared to the Gd-ACA control sample (1.4 ns), all Yb/Er-codoped LnNP-ACA hybrids exhibit significantly shortened singlet lifetimes (0.5–1.1 ns), confirming obvious energy transfer from singlet excitons of ACA molecules to LnNPs. This reduction occurs because Gd^3+^ ions lack suitable energy levels to accept FRET energy, allowing ACA molecules to remain in the excited state for extended periods, whereas the abundant 4f energy levels of Er^3+^ ions provide effective energy acceptor channels for FRET processes. The singlet lifetime exhibits nonmonotonic behavior with increasing shell thickness, initially decreasing from C-ACA (1070 ps) to CS1-ACA (872 ps), reaching a minimum at CS3-ACA (512 ps), and then recovering at CS4-ACA (985 ps). This complex trend reflects the competition between surface passivation effects that enhance Er^3+^ optical activity, as evidenced by the increasingly prominent Er^3+^ transitions in excitation spectra (Figure 3b), particularly the ^4^I_15/2_ → ^4^G_11/2_ transition at 377 nm and the *R* ^–6^ distance dependence of FRET that weakens the energy-transfer efficiency with increasing donor–acceptor separation.

We apply nanosecond TA measurements (Figure 4b), monitoring the triplet–triplet photoinduced absorption at 430 nm (T_1_ → T_*n*_ transition), to reveal pronounced shell-thickness-dependent effects on triplet-state kinetics. All hybrid samples exhibit similar triplet rise times (1.14–1.94 ns), consistent with the fast ISC facilitated by spin-exchange interactions with lanthanide ions, as demonstrated in our previous studies.^32,33^ The minor variations in rise times among samples can be attributed to the heterogeneous molecular environment on NP surfaces, where ACA molecules experience different local environments due to the core composition (Gd^3+^, Yb^3+^, and Er^3+^) versus the Gd^3+^-only shell, along with varying shell thicknesses that alter surface coordination sites. The triplet decay shows shell-thickness-dependent behavior, with all samples exhibiting biexponential decay kinetics extending to microsecond time scales. Compared to the Gd-ACA control sample (298 μs), all Yb/Er-containing samples demonstrate significantly accelerated triplet decay due to TET processes. Notably, the TET efficiencies are determined to be 76%, 72%, 77%, and 73% for C-ACA, CS1-ACA, CS2-ACA, and CS3-ACA, respectively, demonstrating sustained high efficiency for shell thicknesses up to 2.2 nm. However, the TET efficiency drops significantly to 65% for CS4-ACA when the shell thickness reaches 3.0 nm. This result aligns with literature reports showing that the long intrinsic lifetime of triplet excitons and absence of alternative deactivation pathways enable efficient energy transfer even at increased donor–acceptor separations.^33^ The calculated TET efficiency from ACA to Yb/Er-codoped LnNPs is lower than the reported single-doped LnNPs, which reflects competitive energy acceptance. In single-doped systems, all Ln^3+^ serve as uniform triplet acceptors, whereas in our Yb/Er-codoped core, the presence of two acceptor species with different coupling efficiencies effectively dilutes the density of optimal acceptor sites. Nevertheless, TET efficiency remains high, and codoping is essential for achieving desired optical functions, making this trade-off acceptable. While the decay rates follow the expected distance dependence of Dexter-type energy transfer, the sustained high efficiency demonstrates the unique advantage of triplet-mediated energy transfer in these lanthanide–organic hybrid systems.

This study systematically investigates the fundamental trade-off between surface passivation and molecular sensitization in core–shell LnNPs. Through precise shell thickness control (0.8–3.0 nm), we demonstrate that while surface passivation yields dramatic luminescence enhancements, molecular sensitization efficiency exhibits nonmonotonic dependence with optimal performance at ∼0.8 nm. Our spectroscopic investigations reveal that TET efficiency shows a slight decline yet maintains high efficiency (>70%) at relatively thin thicknesses. Importantly, this work addresses a long-standing question in dye-sensitized LnNP systems by demonstrating that thin shells (∼0.8 nm), rather than unshelled cores, provide optimal performance for TET-dominant systems. The optimal optical performance is yielded by balancing the shell passivation, FRET from singlet excitons and TET from triplet excitons. The identification of the optimal shell thickness establishes quantitative design principles for engineering lanthanide–organic hybrid materials with maximum brightness. Our findings transform the optimization approach from empirical methods to rational design strategies based on the fundamental understanding of energy transfer–passivation dynamics. These results provide guidance for developing advanced photonic materials across imaging, energy conversion, and devices.

## Supplementary Material



## Notes

Materials and methods, supporting figures including TEM images, absorption spectra, PL spectra, and energy-level diagrams, and supporting tables including fitted parameters of time-resolved PL decay curves and TA spectra (PDF)
