# Supplementary material for: Balancing Molecular Sensitization and Surface Passivation in Lanthanide-Doped Nanoparticle-Based Organic–Inorganic Nanohybrids
Source: Nano Lett. 2025 Oct 28;25(45):16212–8. doi: 10.1021/acs.nanolett.5c04324 (PMC12616780; doi:10.1021/acs.nanolett.5c04324)
Supplement: Supplementary file 1 [file nl5c04324_si_001.pdf]

Supporting Information

Balancing Molecular Sensitization and Surface Passivation in Lanthanide-Doped Nanoparticle Based Organic-Inorganic Nanohybrids

Zhao Jiang,[1#] Alasdair Tew,[1#] Xinjuan Li,[2] Huangtianzhi Zhu,[1] Yunzhou Deng,[1] Caterina Ducati,[2] Zhongzheng Yu,[1] Akshay Rao[1*]*

[1] Cavendish Laboratory, University of Cambridge, Cambridge, CB3 0HE, United Kingdom
[2] Department of Materials Science and Metallurgy, University of Cambridge, Cambridge, CB3 0FS, United Kingdom

*E-mail address: zy338@cam.ac.uk (Z. Yu), ar525@cam.ac.uk (A. Rao)
#These authors contributed equally: Zhao Jiang, Alasdair Tew.

Materials and Methods

Materials

Gadolinium acetate hydrate ($Gd(CH_3CO_2)_3 \cdot xH_2O$, 99.9%), ytterbium acetate hydrate ($Yb(CH_3CO_2)_3 \cdot xH_2O$, 99.9%), erbium(III) acetate hydrate ($Er(CH_3CO_2)_3 \cdot xH_2O$, 99.9%), sodium hydroxide (NaOH, >98%), ammonium fluoride (NH_4F, 99%), sodium trifluoroacetate (Na(TFA), 98%), trifluoroacetic acid (TFA, 99%), gadolinium(III) oxide (Gd_2O_3, 99.9%), 1-octadecene (ODE, 90%), oleic acid (OA, 90%), 9-anthracenecarboxylic acid (9-ACA, 99%), and all anhydrous solvents (toluene, hexane, ethanol, THF, chloroform, and dichloromethane) were purchased from Sigma-Aldrich. All chemicals were used as received without further purification.

Synthesis of $NaGdF_4$:$Yb_{0.2}$,$Er_{0.02}$ core nanoparticles

0.78 mmol of $Gd(CH_3CO_2)_3$, 0.20 mmol of $Yb(CH_3CO_2)_3$, and 0.02 mmol of $Er(CH_3CO_2)_3$ were added to a 50 mL flask containing OA (6 mL) and ODE (14 ml). The mixture was heated to 140 °C and maintained for 30 min under N_2 flow and then cooled down to room temperature. Subsequently, a methanol solution (8 mL) containing NH_4F (0.96 mmol) and NaOH (0.6 mmol) was added and stirred at 70 °C for 45 min to remove the residual methanol from the reaction mixture. Upon the removal of methanol, the solution was heated to 300 °C and maintained at this temperature under a nitrogen flow for 40 min. The solution was then cooled down to room temperature naturally. The resulting nanoparticles were precipitated out with an addition of ethanol, collected by centrifugation, and finally dispersed in 6 ml of hexane.

$NaGdF_4$ shelling of core nanoparticles

(1) Preparation of gadolinium trifluoroacetate. Gd_2O_3 (5 g) was added to a 50 mL flask. To the flask was added excess water and trifluoroacetic acid (1:1). The mixture was then stirred at 100 °C under reflux until the solution became completely clear and transparent. After cooling to room temperature, this solution was filtered. Finally, the solvents of water and trifluoroacetic acid were evaporated under reduced pressure. A white dry powder of $Gd(TFA)_3$ could be obtained with a yield of >90%.

(2) $NaGdF_4$ shelling. (a) The shell precursor was prepared first. To a 50 mL flask was added Na(TFA) (1.8 mmol, 245 mg), $Gd(TFA)_3$ (1.0 mmol, 496 mg), OA (4 mL), and ODE (6 mL). The solution was evacuated at 110 °C for 30 min and then cooled to room temperature under N_2 flow. (b) To a 50 mL flask was added $NaGdF_4$:$Yb_{0.2}$,$Er_{0.02}$ core NPs (0.3 mmol), OA (8 mL), and ODE (8 mL). The solution was evacuated at 110 °C for 30 min. Then the temperature was increased to 280 °C under N_2 flow. The shell precursor was then added by a syringe pump at a speed of 6.5 mL h^{-1}. Four different amounts (1.5, 3.5, 6.5, 10.0 mL) of shell precursor were added respectively, to give rise to four core-shell NPs with different shell thicknesses. After the injection of shell precursor, the solution was maintained at that temperature for 30 min and then cooled to room temperature. The product was purified three times with ethanol and hexane, and finally dispersed in hexane.

Ligand exchange of NPs with 9-ACA

The concentrations of the whole series of NPs were calibrated by the Yb absorbance at 980 nm (Figure S3) via UV-VIS absorption spectroscopy. Then 1 mL of the OA-capped NPs in hexane were mixed with 0.2 mL 9-ACA solution in THF (0.5 mg/mL). The mixtures were stirred at room temperature for 1 hour. After that, the mixtures were precipitated using ethanol and the LnNP-ACA hybrids were obtained under centrifugation. The products were purified another twice to fully remove the uncoordinated 9-ACA ligands, and finally re-dispersed in hexane for subsequent measurements.

Basic TEM and optical characterizations

Transmission electron microscopy was performed using an FEI Tecnai F20 at 200 kV accelerating voltage. For optical measurements, samples were prepared in a nitrogen-atmosphere glovebox using degassed, anhydrous solvents. Cuvettes were sealed with a PTFE cap, PTFE tape, and parafilm, to ensure the absence of oxygen inside the samples. Steady-state absorption spectra were collected by a Shimadzu UV3600Plus spectrometer. Steady-state photoluminescence spectra were measured using an Edinburgh Instruments FLS1000 spectrometer, equipped with xenon lamp for excitation. The fluorescence decay curves of Er were also measured by FLS1000 spectrometer but using a micfrosecond flashlamp as excitation source. The fluorescence decay curves of 9-ACA were measured using a time-correlated single photon counting (TCSPC) setup, equipped with a 375-nm pulsed laser (pulse width <200 ps, repetition rate: 40 MHz) for excitation and a silicon-based single-photon avalanche photodiode for photon detection. The instrument response function has a lifetime of less than 0.2 ns.

Transient absorption spectroscopy measurement

(1) Picosecond transient absorption (TA) measurements were made using a system from Light Conversion consisting of a PHAROS 1030 nm fundamental laser at a 10 kHz repetition rate, an ORPHEUS OPA for pump generation (355 nm) and the HARPIA TA system for white light generation, delay and capture.

(2) Nanosecond transient absorption measurements were made using a home-built setup consisting of a white light super continuum probe laser from Leukos (DISCO) and a 355 nm pump laser from Innolas (Picolo). Delay between the pump and probe was digitally generated using Highland Technology T560 delay generators. A Streising line camera system was used capture both probe and reference. A function generator from Agilent alongside a Stanford instruments SRS DG645 were used to prepare and shape the initial electronic pulses from triggering the lasers and camera system.

Calculation of triplet energy transfer (TET) efficiency

The TET efficiency of the LnNP-ACA hybrids was calculated using the following formula:

$$\eta_{TET} = 1 - \frac{\tau_{LnNP-ACA}}{\tau_{Gd-ACA}}$$

where $\tau_{LnNP\text{-}ACA}$ and $\tau_{Gd\text{-}ACA}$ are the decay time constants of the $T_1 \rightarrow T_n$ photoinduced absorption of ACA when coupled to LnNP, i.e., in the presence of TET, and Gd control sample, i.e., in the absence of TET, respectively.

Supporting figures

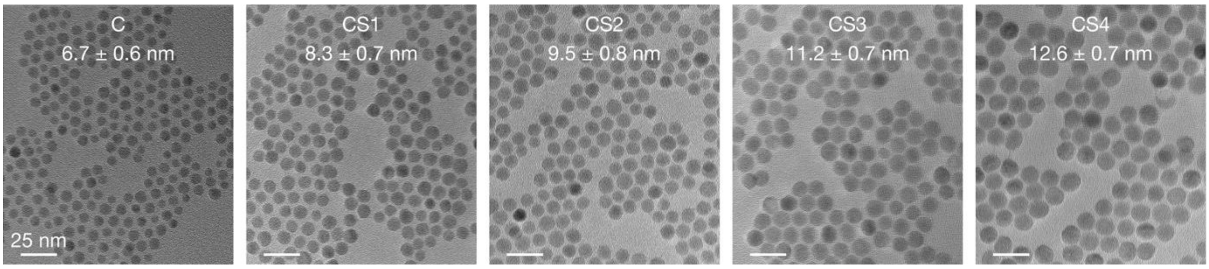

Figure S1. TEM images of the as-synthesized core and core-shell NPs.

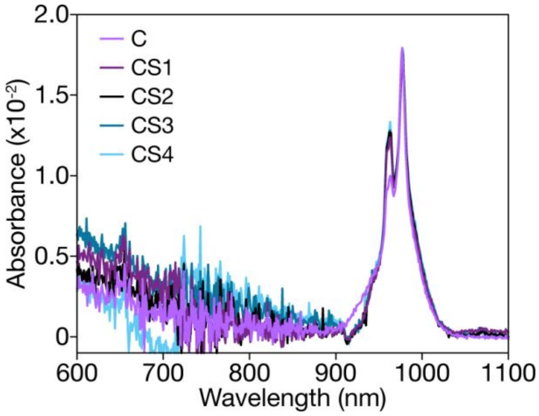

Figure S2. Absorption spectra of core and core-shell LnNPs with calibrated concentrations.

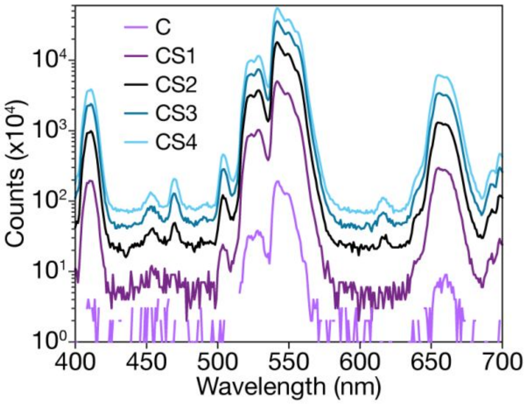

Figure S3. Semi logarithmic plot of upconversion emission spectra of LnNPs under excitation at 980 nm.

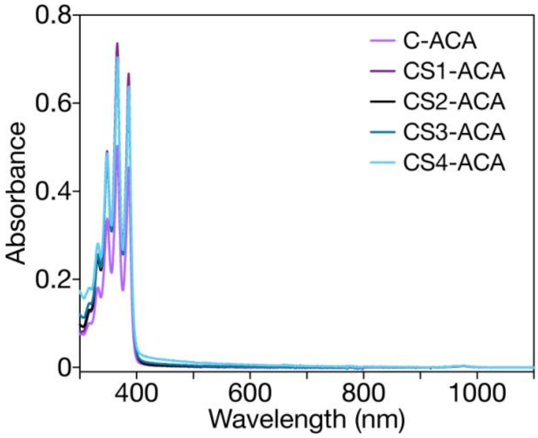

Figure S4. Absorption spectra of LnNPs-ACA samples.

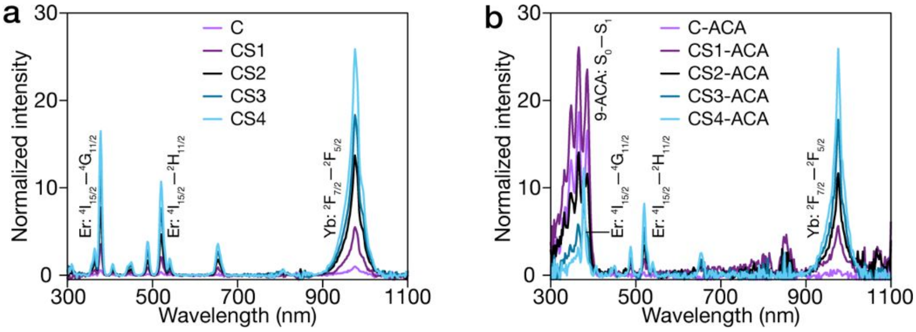

Figure S5. (a) Excitation spectra of pristine core (C) and core-shell (CS1-CS4) LnNPs monitored at Er^{3+} emission (1530 nm). (b) Excitation spectra of LnNP-ACA hybrids monitored at Er^{3+} emission (1530 nm).

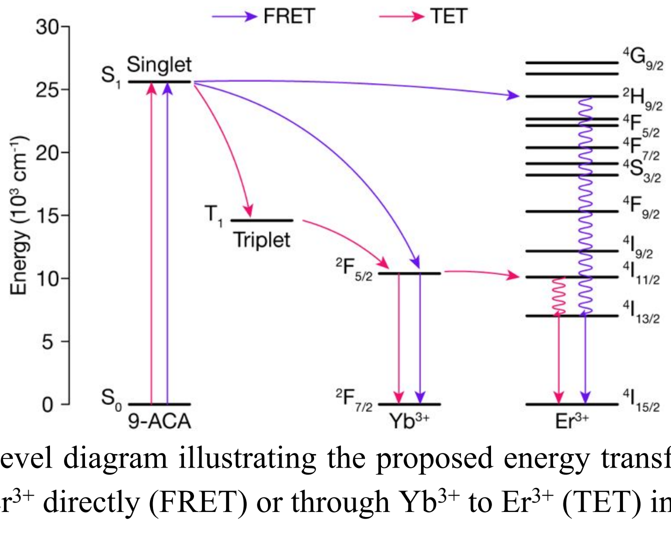

Figure S6. Energy level diagram illustrating the proposed energy transfer pathways from 9-ACA molecules to Er^{3+} directly (FRET) or through Yb^{3+} to Er^{3+} (TET) in the core.

Table S1. Fitted time constants (τ_1 and τ_2) and weights (W_1 and W_2) of the time-resolved PL decay curves showed in Figure 3d.

	9-ACA	Core	CS1	CS2	CS3	CS4
τ_1 (ps)	9528.43	1658.45	1700.84	1833.70	1872.84	1736.51
τ_2 (ps)		7248.34	7223.95	7504.56	7308.75	6823.77
W_1 (%)	100.00	77.34	63.64	64.20	67.26	66.43
W_2 (%)		22.66	36.36	35.80	32.74	33.57

Table S2. Fitted average lifetime and calculated energy transfer efficiency from TA spectra.

	Singlet decay (ps)	Triplet rise (ns)	Triplet decay (ns)	TET efficiency
C-ACA	1070.44	1.94	69990.59	0.76
CS1-ACA	871.50	1.34	83297.70	0.72
CS2-ACA	875.27	1.14	67464.38	0.77
CS3-ACA	511.85	1.75	79199.89	0.73
CS4-ACA	985.24	1.73	104001.11	0.65
Gd-ACA	1438.50	5.68	297766.81	
